# Vitamin D Food Fortification and Nutritional Status in Children: A Systematic Review of Randomized Controlled Trials

**DOI:** 10.3390/nu11112766

**Published:** 2019-11-14

**Authors:** Paula Nascimento Brandão-Lima, Beatriz da Cruz Santos, Concepción Maria Aguilera, Analícia Rocha Santos Freire, Paulo Ricardo Saquete Martins-Filho, Liliane Viana Pires

**Affiliations:** 1 Health Sciences Post-Graduation Program, Department of Medicine, Federal University of Sergipe, Rua Cláudio Batista, S/N, Cidade Nova, Aracaju, 49060-108 Sergipe, Brazil; paulanblima@gmail.com (P.N.B.-L.); saqmartins@hotmail.com (P.R.S.M.-F.); 2 Nutrition Sciences Post-Graduation Program, Department of Nutrition, Federal University of Sergipe, Avenida Marechal Rondon, S/N, Jardim Rosa Elze, São Cristovão, 49100-000 Sergipe, Brazil; cruz14_bia@outlook.com (B.d.C.S.); alicia.nutri@gmail.com (A.R.S.F.); lvianapires@gmail.com (L.V.P.); 3 Department of Biochemistry and Molecular Biology II, Institute of Nutrition and Food Technology, Center of Biomedical Research, University of Granada, Avda. del Conocimiento s/n. Armilla, 18100 Granada, Spain; 4 Instituto de Investigación Biosanitaria IBS.GRANADA, Complejo Hospitalario Universitario de Granada, 18014 Granada, Spain; 5 CIBEROBN (Physiopathology of Obesity and Nutrition Network CB12/03/30038), Institute of Health Carlos III (ISCIII), 28029 Madrid, Spain; 6 Investigative Pathology Laboratory, Federal University of Sergipe, Rua Cláudio Batista, S/N, Cidade Nova, Aracaju, 49060-108 Sergipe, Brazil

**Keywords:** enriched food, child, cholecalciferol, ergocalciferols, dairy products

## Abstract

Children are in the risk group for developing hypovitaminosis D. Several strategies are used to reduce this risk. Among these, fortification of foods with vitamin D (25(OH)D) has contributed to the achievement of nutritional needs. This systematic review aims to discuss food fortification as a strategy for maintenance or recovery of nutritional status related to vitamin D in children. The work was developed according to Preferred Reporting Items for Systematic Reviews and Meta-Analyses (PRISMA) and registered in the International prospective register of systematic reviews (PROSPERO) database (CRD42018052974). Randomized clinical trials with children up to 11 years old, who were offered vitamin D-fortified foods, and who presented 25(OH)D concentrations were used as eligibility criteria. After the selection stages, five studies were included, totaling 792 children of both sexes and aged between two and 11 years. Interventions offered 300–880 IU of vitamin D per day, for a period of 1.6–9 months, using fortified dairy products. In four of the five studies, there was an increase in the serum concentrations of 25(OH)D with the consumption of these foods; additionally, most children reached or maintained sufficiency status. Moreover, the consumption of vitamin D-fortified foods proved to be safe, with no concentrations of 25(OH)D > 250 nmol/L. Based on the above, the fortification of foods with vitamin D can help maintain or recover the nutritional status of this vitamin in children aged 2–11 years. However, it is necessary to perform additional randomized clinical trials in order to establish optimal doses of fortification, according to the peculiarities of each region.

## 1. Introduction

Vitamin D (25(OH)D) is an important nutrient during childhood because of its involvement in bone formation, as well as in the immune system, which can result in higher body needs for this vitamin [1,2,3]. Thus, children are among the groups at risk of developing hypovitaminosis D [4].

Dietary intake is one of the ways to obtain this vitamin, but the food contribution is limited (10% to 20%) [4,5,6]. The main way of obtaining vitamin D is endogenously from sun exposure: ultraviolet B (UVB) rays are absorbed by 7-dehydrocholesterol, producing a thermally unstable compound that is converted in the liver to 25(OH)D, and subsequently converted to the active form 1,25-dihydroxycholecalciferol in the kidneys [3,6,7,8,9].

Even in sunny countries, vitamin D deficiency is observed in different population groups [10,11,12,13]. Considering the issue raised, the fortification of foods with vitamin D is an alternative for wide population coverage for reducing the risk of vitamin D deficiency, and its adoption is increasing worldwide [14,15]. In some countries with high prevalence of vitamin D deficiency and ineffective sun exposure, fortification of foods with vitamin D is compulsory or voluntary, with dairy products being the most frequently fortified foods [16,17,18]. 

There are few clinical trials evaluating food fortification as a strategy to improve or maintain vitamin D nutritional status in child population [14,16,17,18,19], as well as systematic reviews and meta-analysis have evaluated this outcome [20,21]. In this context, the fortification of ready-to-eat foods may contribute to the achievement of the nutritional needs of vitamin D in children, who commonly present a high inadequacy in vitamin D intake [15,22]. Although promising, vitamin D fortification is not yet widely explored by public health policies because more studies are needed to assess the contribution of fortified foods consumption to serum 25(OH)D levels in children and other risk groups of hypovitaminosis D, given the need to obtain information regarding dose, safety, and fortification food vehicle. The objective of this study is to evaluate the available evidence of dairy food fortification as a strategy for maintenance or recovery of nutritional status related to vitamin D in children. 

## 2. Materials and Methods 

This study was conducted following the Preferred Reporting Items for Systematic Reviews and Meta-Analyses statement (PRISMA) [23] (Supplementary 1) and supplemented by guidance from the Cochrane Collaboration Handbook for Systematic Reviews of Interventions [24]. Institutional review board approval and informed consent were not required for this systematic review. A study protocol was designed a priori and was registered in the International prospective register of systematic reviews PROSPERO database (registration number CRD42018052974).

### 2.1. Eligibility Criteria

For the construction of this review randomized clinical trials (RCTs) with children up to 11 years old, who presented data regarding serum or plasma vitamin D (25(OH)D) concentrations at baseline and after the intervention, were considered eligible. The comparators were foods fortified with cholecalciferol or ergocalciferol with non-fortified foods.

Animal or in vitro studies, manuscript published only in summary form, and review studies were excluded. In addition, studies including children diagnosed with diseases that compromised vitamin D metabolism were excluded. To avoid interference from other nutrients, studies who offered fortified foods with vitamin D in conjunction with other nutrients were not considered.

### 2.2. Search Strategy

Searches for RCTs were performed in PubMed, SCOPUS, Bireme, Lilacs, and the website ClinicalTrials.gov from inception to January 2019. A gray-literature search included Google Scholar and OpenThesis. The structured search strategy used the following terms: (child) AND (“vitamin d” OR cholecalciferol OR ergocalciferol OR “fortified food” OR “fortified foods”) (Supplementary 2). The search was limited to studies published in full-text versions, without language restriction. The reference lists of all eligible studies and reviews were scanned to identify additional studies for inclusion. 

### 2.3. Study Selection and Data Extraction

Two evaluators (B.C.S and P.N.B-L) conducted all the selection stages of studies independently, first reading the Titles and Abstracts, and subsequently reading the full studies selected in the first stage. Any disagreement was resolved in conjunction by three evaluators (B.C.S, P.N.B-L, and L.V.P).

To evaluate the agreement between the evaluators in the selection stages of the studies, the kappa coefficient proposed by Landis and Koch [25] was used. The results were classified according to the interval <0 to 1, where <0 = no agreement, 0–0.19 = poor agreement, 0.20–0.39 = fair agreement, 0.40–0.59 = moderate agreement, 0.60–0.79 = substantial agreement, and 0.80–1= almost perfect agreement [25].

The following information was extracted from studies: characteristics of the participants; season of the year; dosage and food matrix used as the fortification vehicle; sun exposure; duration of intervention; and 25(OH)D status. The cut-off points proposed by the World Health Organization were adopted for the classification of BMI (Body Mass Index) for age, with z-score values ≥ −2 and ≤+1 considered normal weight [26].

In the included study that presented the serum 25(OH)D concentration as a graph [16], mean and standard deviation values were extracted using Web Plot Digitizer software version 4.1 (Ankit Rohatgi, Austin, Texas, USA). If the means and standard deviations were not directly reported in the publication, indirect methods of extracting estimates were used [27,28].

### 2.4. Assessment of Risk of Bias

The risk of bias in the studies was independently assessed by two reviewers using the Cochrane Collaboration’s tool for assessing risk of bias in randomized trials [24]. The tool presents seven domains in which random sequence generation, allocation concealment, blinding of participants and professionals, blinding of outcome evaluators, incomplete outcomes, selective outcome reporting, and other sources of bias are evaluated. Other sources of bias were non-descriptions of skin color and frequency of sun exposure of children assessed in the studies. The items contained in this checklist were classified as low risk, high risk, or unclear risk of bias.

### 2.5. Data Synthesis

In order to assess the main outcome, vitamin D intake values were converted into international units per day (µg × 40 = IU) [4] when not provided by the studies. Furthermore, serum concentrations of 25(OH)D were presented in nanomoles per liter (ng/mL × 2.5 = nmol/L) [4]. 25(OH)D levels were analyzed based on change-from-baseline measures [24]. However, due to the heterogeneity observed between the studies, the meta-analysis of the data was not performed. Thus, the results of the studies were reported individually and were not summarized.

The graphical representation of the bias risk analysis was elaborated with Review Manager software 5.3 (The Nordic Cochrane Centre, The Cochrane Collaboration, Copenhagen, Denmark, 2008).

## 3. Results

### 3.1. General Characteristics

**Figure 1:**
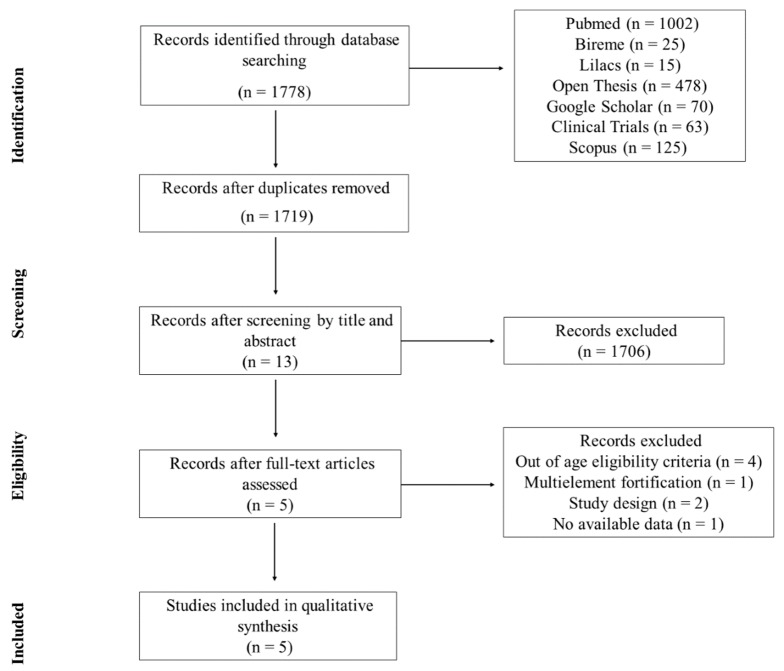
Flowchart of search and selection steps of the studies.

The initial search identified 1778 studies, and, after all the selection stages, five studies met the inclusion criteria and were included in this review [14,16,17,18,19]. The kappa coefficient for two selection stages was, respectively, 0.664 and 0.683, which characterizes a substantial agreement between the evaluators in both stages. The selection stages of the studies are presented in Figure 1.

Of the included randomized clinical trials, four are double-blind type [14,16,17,19], and one is blind [18]. The studies were conducted in four different countries, two being conducted in Canada (latitude > 40° N) [16,17], and the others in Sweden (latitude 55° N e 63° N) [19], Germany (latitude > 50° N) [14], and Mongolia (latitude 48° N) [18].

### 3.2. Risk of Bias

**Figure 2:**
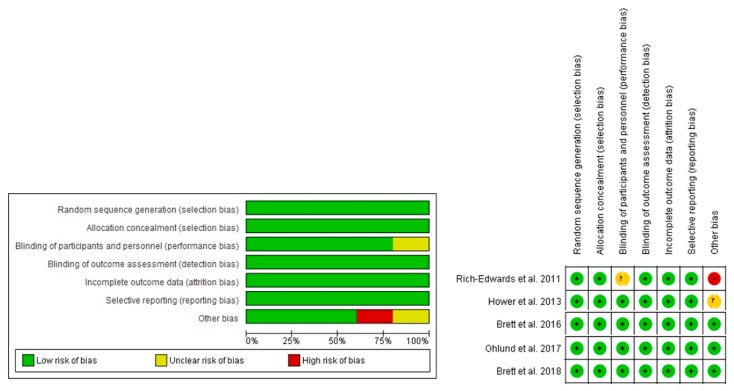
Authors’ judgment about the risk of bias for each included study. Caption: (+) indicates low risk; (−) high risk; and (?) unclear risk.

Regarding the analysis of bias risk (Figure 2), among the five included studies, 100% were classified as low risk of selection bias (random sequence generation). This result was due to the studies using a table of random numbers [14,17] or a computer program [16,18,19] to generate the random sequence. In relation to selection bias (allocation concealment), 100% of the studies presented low risk, while 80% of the studies presented a low risk for the performance bias domain, with only one of the studies lacking sufficient information on the blinding process of participants and professionals [18].

The evaluation of detection, attrition, and reporting bias showed that 100% of the studies presented low risk of bias [14,16,17,19], considering the description of the blinding, the loss of data, and their respective reasons, together with the intention-to-treat analysis, as well as the proposed outcomes were reported, and no bias was observed in the observed effect size.

According to the analysis of other sources of bias (assessment of sun exposure and skin color), 60% of the studies presented low risk [16,17,19]. Only one study did not evaluate such information, being assigned a high risk of bias [18], and another did not clearly describe the method used to evaluate these aspects, being classified as an unclear risk of bias [14].

### 3.3. Sample Characterization

The five studies selected present data on 792 children of both sexes and aged between two and 11 years, distributed in the intervention groups with vitamin D-fortified foods (*n* = 568) and control (*n* = 224). Regarding nutritional status, three studies were conducted with normal weight children according to BMI for age [16,17,18]. Two other studies included in the sample children with normal weight, thinness [14], overweight, and obesity [14,19]. In the studies, no differences were observed between the intervention and control groups in relation to age, sex, and BMI. The general characteristics of the study and the children at baseline are presented in Table 1.

Skin pigmentation was evaluated in four studies, and three of these studies [16,17,19] classified skin pigmentation according to Fitzpatrick’s scale, which considers the existence of six phototypes according to the person’s skin color and response to sun exposure (degree of burning and tanning).

Two studies used a spectrophotometer to measure the individual typological angle and found that more than 50% of the children evaluated had types I to III skin [16,17], whereas Ohlund et al. [19] evaluated the pigmentation of the skin through visual means, characterizing 52% of the children with skin types I to IV in the fair skin group. One of the studies observed that 98% of the children in the sample had a fair skin color, but the methodology used for this classification was not described by the authors [14]. Only the study by Rich-Edwards et al. [18] did not evaluate this information.

The incidence of UVB rays is also a variable that can influence the availability of vitamin D. Two studies were conducted exclusively during a period with no efficient sun exposure (autumn and winter) [18,19], and two other studies also included spring [16,17]; only the study by Hower et al. [14] covered all seasons.

Another relevant aspect to be evaluated is sun exposure. In two studies, no significant differences were observed regarding direct sun exposure among the evaluated children [16,17]. The other studies did not present this information clearly [14,19] or did not evaluate it [18]. Regarding the use of sunscreens, only three studies presented this information [16,17,19].

The children’s vitamin D status at baseline was variable, with the presence of children at risk of deficiency (<30 nmol/L) and of insufficiency (30–49 nmol/L), and with status classified as sufficiency (≥50 nmol/L) of vitamin D (Table 1). However, the experimental design by Hower et al. [14] took into consideration the previous nutritional status as inclusion criterion for the study (>25 nmol/L). The study sample by Rich-Edwards et al. [18] was almost completely composed of children at risk for vitamin D insufficiency and deficiency.

### 3.4. Food Fortification and Intervention Outcomes

Corroborating aspects of vitamin D status, four studies also considered the usual intake of this vitamin. Different methods of assessing food intake were used; among them, semiquantitative food frequency questionnaire (FFQ) [14], short FFQ [19], and the combination of 24-h dietary recall (24HR) and 13-item semiquantitative FFQ [16,17]. The FFQs used were composed of food items with a known contribution to the daily intake of vitamin D (e.g., milk and dairy products, fish, mushrooms) in the study countries. The study by Rich-Edwards et al. [18] was the only one that did not assess the habitual vitamin D intake, only used 24HR to quantify the number of portions of Mongolia’s dairy products consumed daily.

The results showed that at baseline, dietary vitamin D intake was lower than the adequacy recommendations adopted in each study. Dairy products used as vehicles for vitamin D fortification were cheddar cheese, yogurt [16,17], and milk [14,18,19], with concentrations ranging from 42 to 880 IU of vitamin D per serving. Thus, the results were discussed considering the exclusive intervention of the fortified foods offered [14,18,19] or in association with usual daily diet [16,17], for periods ranging from 1.6 to 9 months (Table 2).

By observing the effects of intake of fortified foods on serum vitamin D levels, in three studies, an increase of 25(OH)D levels after the intervention period was observed in all groups receiving fortified foods, whereas the respective control groups did not present alteration of this vitamin in the serum [16,17,19] (Table 3).

In the study by Hower et al. [14], the fortified milk intervention was evaluated during different climatic seasons, showing an increase in the serum 25(OH)D levels after winter in the intervention group that was statistically different from the control group, which presented a reduction in the concentrations of vitamin. In contrast, during the summer, the intervention and control groups remained similar.

Different behavior was observed in the study by Brett et al. [17], in which the fortified dairy products (yogurt and cheddar cheese) promoted maintenance of serum 25(OH)D concentrations during the first 3 months, but at the end of the study (6 months) those concentrations were reduced (Δ = −6.9 nmol/L). On the other hand, in the control group, serum 25(OH)D concentrations reduced at 3 months of the study and remained unchanged until the end of the study (6 months).

Differently, Ohlund et al. [19] observed differences in the 25(OH)D concentrations at baseline according to skin color (dark skin and fair skin) and, with this, the post-intervention data were compared using the analysis of covariance with study groups as a fixed factor and the skin type as a random factor.

The methods used to determine serum 25(OH)D concentrations were liquid chromatography coupled with tandem mass spectrometry (LC-MS/MS) [18,19], high performance liquid chromatography (HPLC) [17], and chemiluminescence immunoassays [14,16]. The quantified forms corresponded to total vitamin D [14,16], and the D2 [18,19] and D3 forms [17,18,19] (Table 3).

In none of the studies, serum 25(OH)D concentrations were above safety limits (>250 nmol/L) in the groups receiving fortified foods, even during the months with abundant sunshine.

## 4. Discussion

This study evaluated the available evidence regarding food fortification as a strategy for maintenance or recovery of vitamin D nutritional status in children. The results of the individual studies suggest that consumption of vitamin D-fortified foods seems to be an important strategy to reduce the prevalence of hypovitaminosis D in this age group, which present greater vulnerability to developing this deficiency. However, due to the clinical and methodological heterogeneity of these studies, a meta-analysis could not be performed.

Previous systematic reviews and meta-analysis sought to evaluate the effect of fortification of foods with vitamin D, demonstrating a significant increase in 25(OH)D concentrations from the consumption of these foods in different population groups [15,20,21,29]. However, when considering only the child group, only one systematic review and meta-analysis has been observed in the literature, with children aged 2–18 years [20]. 

The effects of fortification with vitamin D are reported to be dependent on increased intake of fortified foods, previous vitamin D status, age, body composition, skin pigmentation, sun exposure, geographic aspects, and food intake [14,18,30,31,32]. In addition, for food fortification there is also a need to evaluate the population’s eating habits, especially in the children’s group, so that the used vehicle is routinely present in the food in order to guarantee greater acceptance [14,16].

As noted in the included studies, the use of fortified foods to improve or maintain vitamin D status considered the consumption of milk and dairy products as part of the population’s eating habits. Milk and its products are among the foods often fortified with vitamin D, as they are widely consumed by children, especially in developed countries, and have good bioavailability of the vitamin [33,34].

Dairy products naturally feature in their composition nutrients that act together with vitamin D, thus contributing to the metabolism of this vitamin, as well as in the physiological processes in which it is active [35].

Furthermore, vitamin D is directly related to the calcium present in these foods, in which the 1,25(OH)2D stimulates the intestinal absorption and renal reabsorption of this mineral, as well as acting in the processes of bone mineralization, due its participation in the synthesis of osteocalcin [34]. At the same time, calcium participates in the processes of biosynthesis and regulation of vitamin D, and the reduced content of dietary calcium stimulates the catabolism of 25(OH)D as a result of the elevation of parathormone and 1,25(OH)2D concentrations [34,36]. Despite its sensitivity to light, vitamin D is considered as good micronutrient for fortification [30].

In the countries of origin of the included clinical trials, in areas with restricted sun exposure, it is noted that, although increasing, food fortification is still not widely adopted. In Canada, where two of the five included studies were conducted [16,17], only liquid cow’s milk (35–45 UI/100 mL), and margarine (530 UI/100 g) must be fortified in accordance with current legislation [4,31]. In Sweden, the Swedish National Food Agency has established recent changes in fortification policy by expanding the list of foods and the levels to be fortified [37]. However, in Germany and Mongolia there is no food fortification policy, although they are located in a region with a latitude greater than 45° N and have a high prevalence of vitamin D deficiency [14,18,38].

Different responses to food fortification at serum vitamin D concentrations can be observed depending on previous nutritional status [16,18]. Children with poor or insufficient vitamin D status had a significant increase in serum 25(OH)D concentrations after the intervention [14,16,18,19], which was not identified in children with sufficient status [17]. This is because the evidence of the relationship between vitamin D intake and serum concentrations is non-linear in nature; that is, the intake of vitamin D has less impact on their serum concentrations when they are at high or sufficient levels [4,39]. Considering this, Aloia et al. [39] showed that in order to alter serum vitamin D concentrations above 50 nmol/L, there would be a need for a higher intake of vitamin D when compared to the amount necessary to increase serum vitamin D levels under 50 nmol/L.

When circulating 25(OH)D concentrations are present in sufficiency, a portion of this vitamin can be transferred to body stores once saturation of the vitamin D 25-hydroxylase (CYP2R1)—the enzyme responsible for vitamin D hydroxylation in the liver—occurs [16,17]. The aspects related to the tissue distribution of vitamin D in children are not widely known; however, studies indicate that vitamin D intake is associated with an increase in lean mass and bone mineral density in healthy children [17,36]. 

The discussion of dose-response of fortification at 25(OH)D concentrations is little explored. Brett et al. [16] observed, by linear regression analysis, that each 100 IU per day increase in vitamin D intake by through fortified foods resulted in an increase in serum 25(OH)D concentrations of 0.6–10 nmol/L in the children. A similar result could be observed in the study by Rich-Edwards et al. [18] in which the increase in serum concentrations of 25(OH)D was 15 nmol/L per 100 IU of vitamin D intake. In the only systematic review and meta-analysis performed in children (2–18 years) to date, the authors sought to investigate the effect of vitamin D interventions (fortified foods, supplements, bolus injections) on vitamin D status. In this study, the authors observed greater results in the mean change in serum 25(OH)D per 100 IU vitamin D/d in trials only using fortified food (*n* = 7; 6.9 nmol/L; 95% CI: 3.7, 10.0 nmol/L; *I*^2^ = 99.9%) than trials that offered daily supplements (*n* = 15; 2.9 nmol/L; 95% CI: 2.4, 3.5 nmol/L; *I*^2^ = 56%) (*p* = 0.001), and bolus injections (*n* = 2; 2.3 nmol/L; 95% CI: 0.9, 3.9 nmol/L; *I*^2^ = 0%) (*p* = 0.04). The authors demonstrated that the serum 25(OH)D response to vitamin D intake differed on the basis of baseline status, intakes, and delivery mode, but not age, sex, or latitude [20]. However, the overall results should be used cautiously due to the high level of heterogeneity observed.

A meta-analysis published by Black et al. [15], performed with adults, showed similar results. The increase of 1.2 nmol/L (95% CI: 0.72, 1.68; *I*^2^ = 89%) was observed in the 25(OH)D concentrations for each 40 IU day of vitamin D ingested from fortified foods. In the paper by O’Donnell et al. [29], the combining results of four trials that offered vitamin D fortified-milk (*n* = 466 individuals; 138–800 IU vitamin D/day) also demonstrated an increase in the 25(OH)D concentrations in adults (15.63 (12.79, 18.48) nmol/L; *I*^2^ = 0.0%; *p* = 0.77).

From the evaluated results, it is not possible to determine the optimal fortification dose, since changes in 25(OH)D concentrations were observed in almost all trials in different populations. However, the dose range of vitamin D used in fortification of foods from included studies (300–800 IU/day) were effective in maintaining or recovering vitamin D status in children. Besides, the optimal dose could be dependent on the characteristics of the population targeted, including those with vitamin D deficiency.

The intervention period, another relevant aspect for the study outcome, varied among the studies. In some of the included studies, this variable was chosen because it corresponds to the length of the season of interest. Even so, all studies exceeded the considered half-life of 25(OH)D which is between 2 and 3 weeks [40]. Vitamin D3 obtained orally has a circulatory peak of 12 h, returning to basal concentrations within 7 days, even when given high doses [41]. Chronic vitamin D supplementation is shown to be the best alternative for promoting gradual increase and sustaining constant 25(OH)D concentrations [20,42], reaching a steady state in healthy adult subjects at 3 months, approximately [43,44]. More importantly, there is not enough evidence on vitamin D metabolism in children [45,46].

In children, deficient vitamin D status is the main cause of rickets, a disorder characterized by disturbances in bone growth and skeletal abnormalities, besides negatively influencing cognitive development, hormone formation, and immune function [32,47,48]. It is important to consider that the early years of childhood are characterized by high rates of growth velocity, requiring specific values of energy and micronutrients such as vitamin D [32,49].

In addition, other variables that may affect the vitamin D bioavailability should be considered. Body composition is an important aspect for understanding the metabolism of this vitamin because adiposity has been inversely associated with 25(OH)D concentrations by acting on vitamin D storage, increasing its clearance [4,17,50,51]. Thus, children with excess body fat may present an increased risk for the development of 25(OH)D deficiency, requiring two to three times higher concentrations of vitamin D to meet bodily needs [50].

Overall, studies have shown that fair-skinned individuals have higher levels of 25(OH)D compared to darker skinned individuals [6,52], a fact that could be observed in one of the included studies [19]. The variation in skin pigmentation depends on the type and amount of melanin generated, as well as on the activity of keratinocytes, which are responsible for the sequestration and degradation of melanin [53]. Thus, the greater the pigmentation of the skin, the lower the vitamin D production, since the melanin absorbs the UVB radiation that would act in the synthesis of this vitamin [36].

Geographical location and climatic characteristics also directly influence the synthesis of vitamin D in the skin, with low or absent synthesis observed during most of the winter in places with latitudes above 33° [50]. In this study, all studies included were performed in countries with a latitude greater than 35°, and the individuals studied presented mostly fair skin (Fitzpatrick classification I to III). Since during a part of the year the UVB radiation in these places is not sufficient to activate the endogenous synthesis and consequently maintain the status of this vitamin [50], food fortification takes on greater importance in the context of public policy.

It is known that diet has a lower participation in 25(OH)D concentrations [47]. This is because the reduced variety of vitamin D food sources means they cannot be part of the eating habits of individuals, especially children [14,18,33]. Among the available food sources, we can mention fatty fish, cod liver oil, mushrooms, egg yolk, and liver steak [4,29,33]. In addition, regular consumption of vitamin D-fortified foods can provide better results in maintaining sufficient vitamin D status compared to other strategies such as seasonal capsule supplementation or the supply of high-dose vitamin D acutely [14,16]. 

In addition, methods of serum 25(OH)D assessment have a strong influence on the diagnosis of vitamin D status. There is no consensus regarding the reference standard due to susceptibility to problems in the pre-analytical, analytical, and post-analytical phases. Immunoassays are dependent on the specificity of the antibodies used, not being able to identify the 3-epi-25(OH)D molecule. The HPLC method has been replaced by the LC-MS/MS method, currently considered the gold standard because of its higher sensitivity [4,54]. It should be noted the included studies showed clinical and methodological heterogeneity that could not be sufficiently explored in the subgroup analysis and could produce clinically inconsistent results. Thus, the food fortification strategy is promising, but there is a need to evaluate the dose-response of vitamin D-fortified foods in vitamin D status, considering all aspects involved in bioavailability, especially population-wide implementation.

This is the first systematic review that addresses the effects of vitamin D-fortified food intake by children aged 2–11 years, taking into account the other factors that influence vitamin D metabolism, such as latitude, sun exposure, skin color, use of sunscreen, and usual dietary intake. Furthermore, this review included studies that offered fortified foods exclusively with vitamin D in order to minimize additional factors that could interfere in the bioavailability of this vitamin.

Regarding limitations of the present study, it is possible to mention the low number of studies available in the literature that offered vitamin D-fortified foods for children. Thus, this study recommends further randomized clinical trials of this age group to increase evidence and identify the optimal dose for vitamin D food fortification. Moreover, it should be emphasized that the habitual consumption of the evaluated groups should be considered, even in the face of the limitations inherent in the methods used to evaluate habitual nutrient intake. 

## 5. Conclusions

The results of the individual studies suggest that the fortification of foods with vitamin D can be used for maintaining or recovering the vitamin’s nutritional status in children aged 2–11 years. In addition, the intake of 300–880 IU of vitamin D per day through the consumption of fortified foods appears to be safe under the conditions studied, with no increase in serum 25(OH)D concentrations above the tolerable limits. However, due to the insufficient number and heterogeneity between the studies, a meta-analysis evaluating the outcomes of interest was not performed, and a pragmatic recommendation on the fortification of foods with vitamin D is not possible. It is necessary to carry out additional randomized clinical trials in order to increase the strength of evidence, and to establish ideal doses of fortification at the population level.

## Figures and Tables

**Table 1:** General characteristics of the studies and the children at baseline.

Author	Country	Duration of Study/Season	Group	No. of Children	Age (Years)	Vitamin D Status (*n*)		BMI for Age Classification ^ǂ^	Skin Phototype (*n*) ^§^
						Deficiency and Insufficiency	Sufficiency		
Rich-Edwards et al. [18]	Mongolia	January to March/Winter	Mongolian Milk	140	10.0 ± 1.0	NA	1	Normal weight	NA
			UHT USA Milk	137			5		
			Control	101			1		
Hower et al. [14]	Germany	November to July/Fall to Summer	Intervention	46	3.8 (2.0–6.8)	21	25	Thickness, normal weight, overweight and obesity	Light skin: 78 ^¥^
			Control	34	3.7 (2.0–6.2)	19	15		Dark skin: 2 ^¥^
Brett et al. [16]	Canada	January to April/Winter to mid-spring	EAR	27	4.9 ± 2.1	7	20	Normal weight	Phototype I to III: 43 Phototype IV to VI: 34
			RDA	26	5.3 ± 2.0	4	22		
			Control	24	5.0 ± 1.8	7	17		
Ohlund et al. [19]	Sweden	November to March/Fall to Winter	10 µg	80	6.3 (6.2;6.7)	NA	47	Normal weight, overweight and obesity	Phototype I to IV: 108 Phototype V to VI: 98
			25 µg	86	6.3 (6.2;6.4)		54		
			Control	40	6.3 (6.1;6.5)		20		
Brett et al. [17]	Canada	October to March/Fall to Winter	Intervention	26	5.0 ± 1.8	2	24	Normal weight	Phototype I to III: 34 Phototype IV to VI: 17
			Control	25	5.4 ± 2.0	2	23		

Data presented as mean ± standard deviation, median (minimum–maximum) or mean (95% confidence interval); ǂ Classification of BMI for age according to the World Health Organization [26]; § Skin phototypes classified by the authors of the studies using the Fitzpatrick scale or ¥ by method not informed. BMI: Body Mass Index; EAR: Estimated Average Requirement; RDA: Recommended Dietary Allowances; UHT: Ultra-High Temperature; USA: United States; NA: Data not available in the papers.

**Table 2:** Characteristics of interventions and food fortification of the studies.

Author	Duration of Study (Months)	Group	Food/Portion Size	Vitamin D Content in Food	Total Vitamin D (IU/Day)
Rich-Edwards et al. [18]	1.6	Mongolian milk	Mongolian milk/710 mL	100 IU/236 mL	300
		UHT USA milk	UHT USA milk/710 mL	100 IU/236 mL	300
		Control	Non-fortified milk/710 mL	NA	NA
Hower et al. [14]	9	Intervention	Fortified milk /350 mL	114 IU/100 mL	400
		Control	Non-fortified milk /350 mL	1.2 IU/100 mL	4.2
Brett et al. [16]	3	EAR	Yogurt/186 mL	42 IU/ 93 mL	400 ¥
			Cheddar cheese /21 g	200 IU/21 g	
		RDA	Yogurt/186 mL	125 IU/93 mL	600 ¥
			Cheddar cheese/21 g	200 IU/21 g	
		Control	Non-fortified yogurt/186 mL	15 IU/93 mL	140–195 ¥
			Non-fortified cheddar cheese/21 g	NA	
Ohlund et al. [19]	3	10 µg	UHT milk/200 mL	480 IU/200 g	480
		25 µg	UHT milk/ 200 mL	880 IU/200 g	880
		Control	Non-fortified UHT milk/200 mL	80 IU/200 mL	80
Brett et al. [17]	6	Intervention	Yogurt/186 mL Cheddar cheese/33g	Yogurt: 150 IU/93 mL Cheddar cheese: 300 IU/33 g	400 ¥
		Control	Non-fortified yogurt/186 mL Non-fortified cheddar cheese/33 g	NA	140–195 ¥

¥ Studies have counted the usual intake of 110–165 IU of vitamin D from foods routinely consumed in the total daily intake. EAR: Estimated Average Requirement; NA: Data not available in the papers; RDA: Recommended Dietary Allowances; UHT: Ultra-High Temperature; USA: United States.

**Table 3:** Effect of consumption of the vitamin D-fortified foods in children.

Author	Methods of Vitamin D Assessment	Group	25(OH)D (nmol/L)			Δ Change (nmol/L)	
			Baseline	End Point			
Rich-Edwards et al. [18]	LC-MS/MS	Mongolian milk	20.0 ± 10.0 ^a^	50.0 ± 15.0 ^b,#^		30.0 ± 13.2	
		UHT USA milk	25.0 ± 12.5 ^a^	72.4 ± 25.0 ^b,#^		47.4 ± 21.7	
		Control	20.0 ± 10.0 ^a^	20.0 ± 10.0 ^a^		0 ± 10.0	
Hower et al. [14]	CLIA	Intervention	53.7 ± 20.6 ^a^	After winter 62.0 ± 25.8 ^b,#^	Summer 69.0 ± 13.6 ^b^	After winter 8.3 ± 23.6	Summer 15.3 ± 18.1
		Control	46.0 ± 21.2 ^a^	After winter 34.0 ± 18.6 ^b^	Summer 68.5 ± 13.0 ^c^	After winter −12.0 ± 20.0	Summer 22.5 ± 18.5
Brett et al. [16]	CLIA	EAR	59.7 ± 13.0 ^a^	64.2 ± 9.7 ^b,#^		4.5 ± 11.7	
		RDA	60.9 ± 10.1 ^a^	64.1 ± 11.8 ^b,#^		3.2 ± 11.0	
		Control	58.6 ± 14.5 ^a^	56.1 ± 11.9 ^a^		−2.5 ± 13.4	
Ohlund et al. [19]	LC-MS/MS	10 µg	56.0 ± 18.3 ^a^	69.0 ± 9.1 ^b,#^		13.0 ± 15.8	
		25 µg	58.0 ± 21.3 ^a^	82.0 ± 14.2 ^b,#^		24.0 ± 18.8	
		Control	49.0 ± 19.4 ^a^	50.0 ± 14.5 a		1.0 ± 17.5	
Brett et al. [17]	HPLC	Intervention	65.3 ± 12.2 ^a^	3 months 64.7 ± 12.2 ^a,#^	6 months 58.4 ± 8.7 ^b^	3 months −0.6 ± 12.2	6 months −6.9 ± 10.9
		Control	67.5 ± 15.1 ^a^	3 months 58.3 ± 15.3 ^b^	6 months 56.6 ± 13.9 ^b^	3 months −9.2 ± 15.2	6 months −10.9 ± 14.5

Data presented as mean ± standard deviation; # denotes significant difference in relation to the control group; different superscript letters denote significant differences within the group over time. 25(OH)D: serum vitamin D concentration; CLIA: chemiluminescence immunoassay; EAR: Estimated Average Requirement; HPLC: High Performance Liquid Chromatography; LC-MS/MS: liquid chromatography coupled with tandem mass spectrometry; RDA: Recommended Dietary Allowances; UHT: Ultra-High Temperature; USA: United States.

## Supplementary Materials

The following are available online at https://www.mdpi.com/2072-6643/11/11/2766/s1, Supplementary 1: PRISMA 2009 checklist; Supplementary 2: Structured search strategy used for systematic search on databases.
