# Supplementary material for: Vitamin D Food Fortification and Nutritional Status in Children: A Systematic Review of Randomized Controlled Trials
Source: Nutrients. 2019 Nov 14;11(11):2766. doi: 10.3390/nu11112766 (PMC6893768; doi:10.3390/nu11112766)
Supplement: Supplementary file 1 [file nutrients-11-02766-s001.zip › nutrients-615741-supplementary/Supplementary 2.docx]

**Structured search strategy used for systematic search on databases**

(("child"[MeSH Terms] OR "child"[All Fields]) AND ("vitamin d"[All Fields] OR ("cholecalciferol"[MeSH Terms] OR "cholecalciferol"[All Fields]) OR ("ergocalciferols"[MeSH Terms] OR "ergocalciferols"[All Fields]) OR "fortified food"[All Fields] OR "fortified foods"[All Fields])) AND (Clinical Trial[ptyp] AND "humans"[MeSH Terms])
